# Single molecule counting and assessment of random molecular tagging errors with transposable giga-scale error-correcting barcodes

**DOI:** 10.1186/s12864-017-4141-4

**Published:** 2017-09-21

**Authors:** Billy T. Lau, Hanlee P. Ji

**Affiliations:** 1 Stanford Genome Technology Center, Stanford University, Palo Alto, CA 94304 USA; 2 Division of Oncology, Department of Medicine, Stanford University School of Medicine, Stanford, CA 94305 USA

**Keywords:** Transcriptome, RNA-Seq, Molecular barcoding

## Abstract

**Background:**

RNA-Seq measures gene expression by counting sequence reads belonging to unique cDNA fragments. Molecular barcodes commonly in the form of random nucleotides were recently introduced to improve gene expression measures by detecting amplification duplicates, but are susceptible to errors generated during PCR and sequencing. This results in false positive counts, leading to inaccurate transcriptome quantification especially at low input and single-cell RNA amounts where the total number of molecules present is minuscule.

To address this issue, we demonstrated the systematic identification of molecular species using transposable error-correcting barcodes that are exponentially expanded to tens of billions of unique labels.

**Results:**

We experimentally showed random-mer molecular barcodes suffer from substantial and persistent errors that are difficult to resolve. To assess our method’s performance, we applied it to the analysis of known reference RNA standards. By including an inline random-mer molecular barcode, we systematically characterized the presence of sequence errors in random-mer molecular barcodes. We observed that such errors are extensive and become more dominant at low input amounts.

**Conclusions:**

We described the first study to use transposable molecular barcodes and its use for studying random-mer molecular barcode errors. Extensive errors found in random-mer molecular barcodes may warrant the use of error correcting barcodes for transcriptome analysis as input amounts decrease.

**Electronic supplementary material:**

The online version of this article (10.1186/s12864-017-4141-4) contains supplementary material, which is available to authorized users.

## Background

RNA-Seq measures gene expression by counting aligned sequencing reads [1, 2] and can quantitate bulk and individual cell transcriptomes [3]. To improve counting accuracy, ‘molecular barcodes’ have been recently introduced to distinguish PCR duplicates from new molecules [4–9]. Molecular barcodes link sequence reads to a single originating nucleic acid molecule by attachment to the sequencing adapter, and enables the tracking of single molecules through a sequencing assay. One barcode type is composed of random nucleotides (‘random-mers’); each unique random-mer tag assists in the identification of single nucleic acid molecules [7, 8]. Alternatively, rationally designed sequences can be used to identify individual molecules [4, 5, 9].

PCR amplification and sequencing chemistry introduce barcode errors that adversely affect molecular barcode analysis [8]. For example, an error in a molecular barcode results in a false positive count of a transcript molecule. While random-mer barcodes are currently the most popular type of molecular barcode, their inherent randomness means they cannot encode any information about their identity. As such, sequence errors may be difficult to distinguish from genuine molecular barcode sequences. This has profound consequences when studying transcriptomes derived from only a small number of total transcript molecules, such as from sparing amounts of bulk RNA or from single cells.

Several methods exist to address errors in random-mer molecular barcodes. One robust random-mer error correction scheme relies on finding reads that contain the reverse complement of a barcode; however, this requirement greatly reduces the yield of informative data [8, 10]. Random-mers can also be filtered by abundance, where rare molecular barcodes due to PCR or sequencing errors are excluded [11]. However, this requires a high saturation of the sequencing library and reduces the amount of useful sequencing power. More commonly, edit distance or Hamming distance metrics, are used to group together minimally distorted molecular barcodes [12–14]. Recently, studies have modified random-mer molecular barcode designs to improve nucleotide balance [15]. Software packages that employ network-based methods for detecting and correcting random-mer based errors have also been recently demonstrated [16].

Rationally designed molecular barcodes in contrast are error-resistant, but the synthesis of individual barcodes is limited to the order of hundreds of oligonucleotides; this is several orders of magnitude lower than random-mer barcodes thus making molecular assignment challenging [4, 5, 9]. Unique alignment positions are thus used alongside molecular barcodes to facilitate molecule identification, but the performance varies greatly between aligners [17]. Overall, there is a need for robust molecular barcodes that combines large diversities found in random-mer barcodes with error-correcting capabilities of rationally designed molecular barcodes.

As a solution, we developed a rational molecular barcoding strategy with giga-scale diversity (i.e. billions) and a novel error-correction strategy that is robust against polymerase and sequencing errors. These exponentially-expanded barcodes **(EXBs)** are produced with an enzymatic molecular assembly method, increasing the diversity of rationally designed barcodes by six orders of magnitude. A transposase introduces the EXBs into double stranded cDNA molecules resulting in a paired-end inline barcode structure, and enables the production of DNA sequencing libraries from small amounts of nucleic acid molecules [18]. While barcoded Tn5 transposon cassettes have been used for tagging and mutagenesis inside bacterial genomes [19], we believe that EXBs are the first demonstration of in vitro transposition of molecular barcodes for next-generation sequencing library preparation.

In this study, we validated this approach by characterizing “ground truth” transcriptome libraries tagged with EXBs. To the best of our knowledge, we also provided the first study that investigates the extent of sequence errors in random-mer molecular barcodes by utilizing an inline barcode control. Although molecular counting is typically used in single-cell RNA-Seq, extensive technical variability and cellular heterogeneity would appear as confounding factors when assessing counting performance. Here, we instead focused on well-characterized bulk RNA samples that have been extensively examined over decades of consortium studies. We showed improved molecular counting performance when starting with picogram amounts of cDNA. We demonstrated that random-mer molecular barcodes suffer from nucleotide errors that are difficult to resolve without improved tagging methods but can be recovered with EXBs.

## Results

### Overview of EXB-based molecular barcoding

**Fig. 1:**
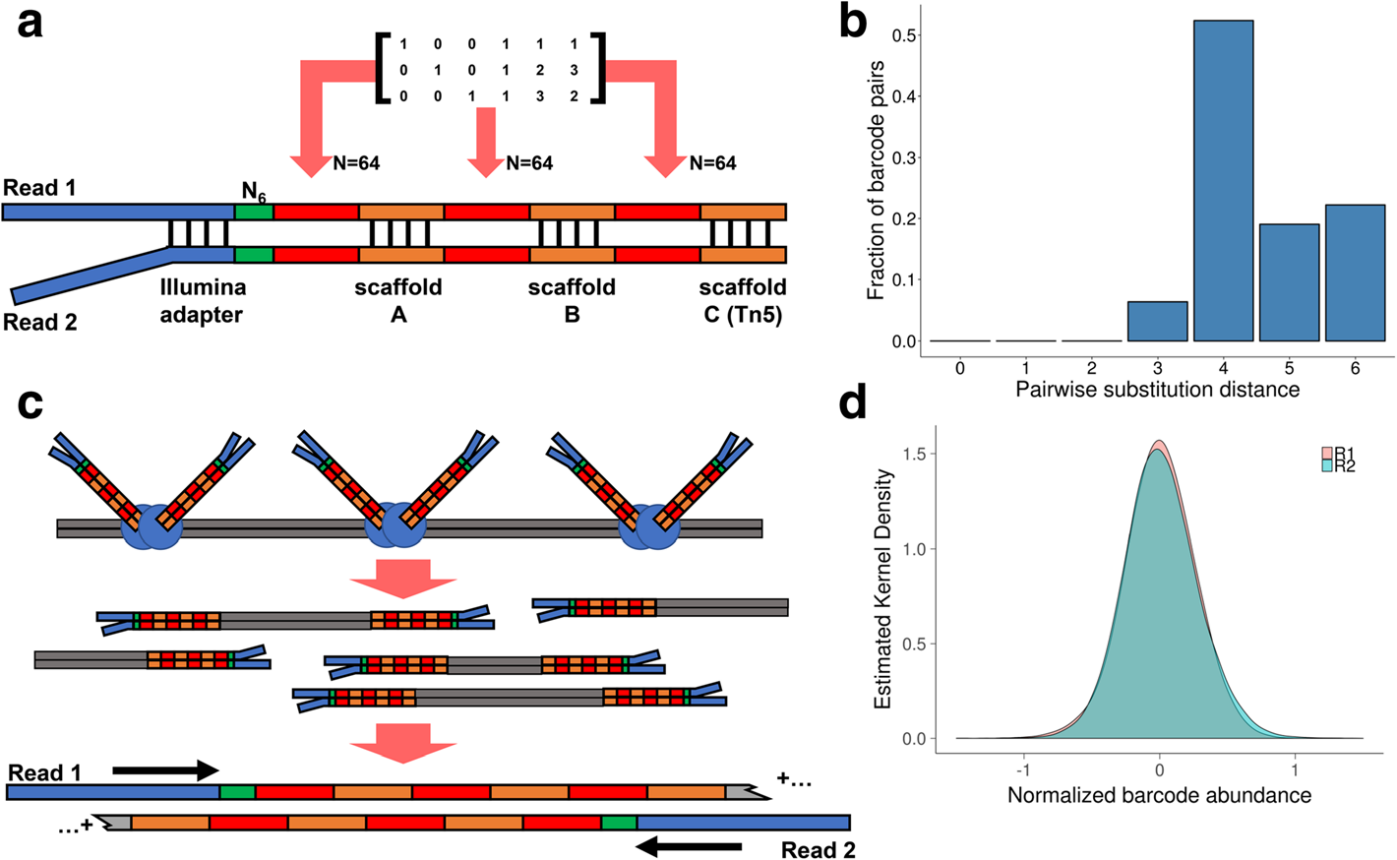
Overview of EXB-based molecular barcoding. **a** Structure of the EXB adapter. The adapter consists of a paired-end Y-adapter structure followed by a 6 bp random nucleotide sequence and three rationally designed 6 bp barcode subunits separated by distinct scaffold sequences. The 6 bp barcode subunits are random combinations of 64 possible sequences as output from the linear generator matrix as shown. The Tn5 transposase recognition sequence at the end of the adapter allows for the generation of sequencing libraries via in vitro Tn5 transposition. **b** Edit (substitution) distance metrics for all possible 6 bp barcode pairs. Over 93% of pairwise comparisons between barcodes have an edit distance greater than 4. **c** Schematic of in vitro transposition of EXBs. Tn5 transposase loaded with EXB adapters are incubated with double stranded cDNA. A gap-fill repair reaction then generates paired-end EXB sequencing libraries. After PCR, EXBs are read as inline barcodes, after which the insert sequence is read. **d** Single-end abundance of EXBs. Single-ended EXB identities were measured by pooling one million reads of each library

EXBs use a computationally designed set of highly error-resistant barcodes followed by enzymatic assembly process to generate high diversity. A linear code in the form of a generator matrix produces a small set of barcodes with high pairwise edit distances **(**Fig. 1a, Additional file 1 **:** Figure S1a**)**. Linear codes are a type of error correction strategy where redundant “bits” corresponding to mathematical linear combinations of the original signal are transmitted in tandem. For example, Hamming codes are a type of binary linear code commonly used in computing systems to control for errors in data transmission and storage [20]. DNA barcodes generated with this strategy are robust; artefactual base changes can be detected and corrected for the original barcode. Using this strategy, shorter barcodes are lengthened with redundancy bases. From all possible 3-mer combinations, we used an optimal quaternary linear generator matrix to product a corresponding set of 64 6-mer barcodes; over 90% of pair-wise edit distances are 4 or greater **(** Methods **,** Fig. 1b **,** Additional file 1 **:** Table S1).

Overcoming the limits of rational barcode numbers, our method generated exponentially large numbers of unique, rationally-designed barcodes. Pooled barcode-scaffold oligonucleotides are combinatorially assembled with an enzymatic extension-ligation reaction **(**Additional file 1 **:** Figure S2**,** Methods). EXBs are comprised of three subunits, each of which comprising one of 64 previously generated 6-mer barcodes. In total, 194 oligonucleotides corresponding to the three subunit regions and two common oligonucleotides are required **(**Additional file 2 **:** Table S2), which results in 262,144 (64^3^) molecular barcodes for each single-end read. This led to the generation of 69 billion (262,144^2^ = 68,719,476,736) paired-end EXBs. A Tn5 transposase introduces EXBs into double-stranded cDNA molecules [21, 22]; EXBs are read as inline DNA elements before the cDNA sequence (Fig. 1c). The total length of the inline EXB is 71 bp, which allows for 80 bp bases of insert from a 2x151bp paired-end assay. Gel electrophoresis showed that the EXB adapter is estimated to be at least ~60-70% pure (Additional file 1: Figure S1b), although downstream transposase loading and subsequent library amplification by PCR effectively selected for full length adapter structures.

### Technical performance of EXBs on cDNA standards

We generated EXB-based libraries with 10 ng, 1 ng, and 100 pg of brain and human cDNA from the Microarray Quality Control project **(MAQC)** [23] **(**Additional file 1 **:** Table S3-S5). This protocol is adapted from an established protocol for the ‘tagmentation’ of double-stranded cDNA [22], and is the first demonstration of the inclusion of molecular barcodes in the ‘tagmentation’ reaction. To filter out EXB chimeras due to self-tagmentation, we used size selection on a Pippin Prep instrument to remove PCR products less than 500 bp long (Additional file 1: Figure S1c). We counted the EXB sequences on each read end and observed 99% of all single-end EXBs are within a logarithm of the median abundance, corresponding to coefficient of variations of 0.66 and 0.89 for read 1 and read 2 respectively **(**Fig. 1d **)**.

The analysis of EXB-tagged sequence reads used an alignment-free approach for counting molecules. A linear check decoder matrix identified the closest matching barcode sequence along the adapter region **(** Methods **,** Additional file 1 **:** Figure S3a). A check matrix is derived from the generator matrix, and detects the presence of “bits” that are inconsistent with the mathematical formula used in the generation of codes. In the context of this study, the erroneous barcode positions for each read are marked, then matched to the closest possible barcode with only the erroneous positions changed (Additional file 1: Figure S3b).

**Fig. 2:**
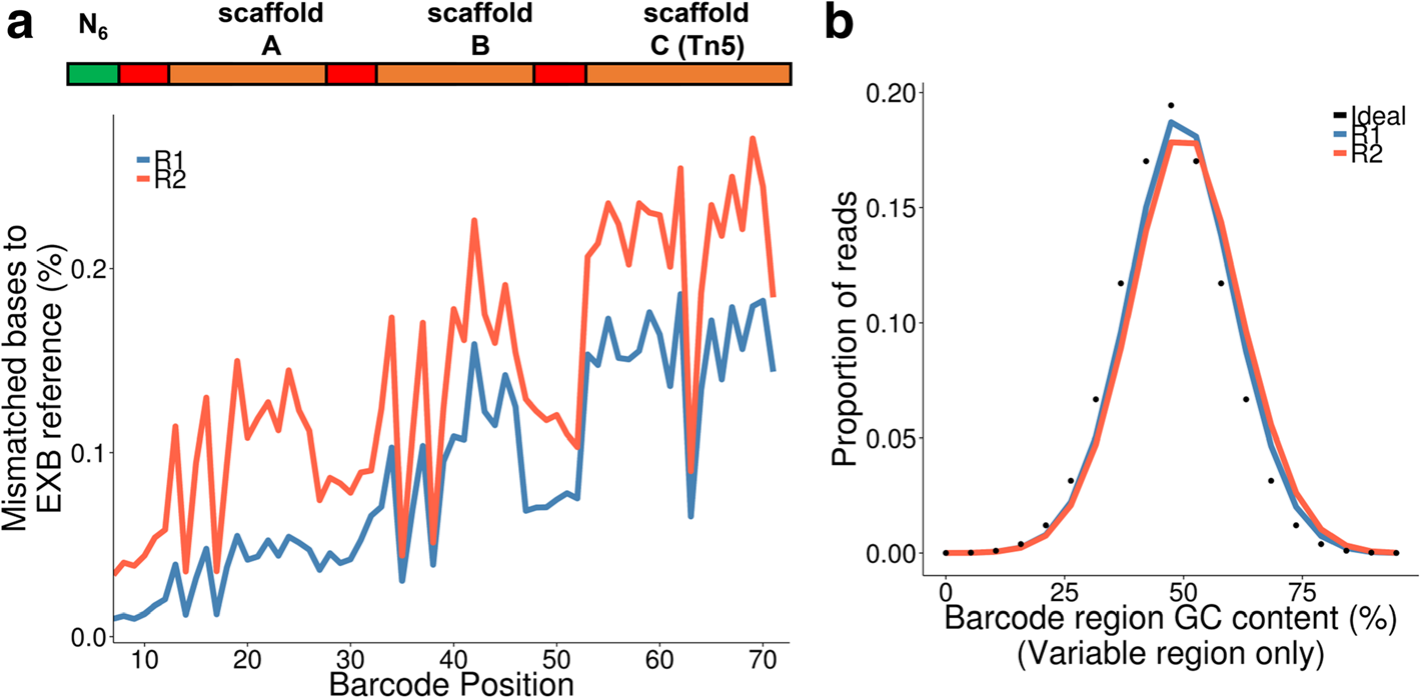
Experimental characterization of EXBs. **a** Error rate between single-end EXB sequence and best decoder matrix match. One million reads from each bulk sample type and input amount are pooled. The mean mismatch rate between the experimentally-derived EXB sequence and the best decoder matrix match is plotted as a percentage of all sequenced EXB bases against the base position along the EXB structure. **b** GC bias of single-end EXBs. One million reads from each library were pooled. The mean GC content for both read 1 and 2 of the barcode containing region (18 bp total) is calculated for each observed single-end EXB. An ideal GC-content distribution corresponding to a uniform distribution of all single-end EXBs is shown as dots. *t*-test results: *p* = 1 (ideal versus read 1), and *p* = 1 (ideal versus read 2), indicating non-significant differences between distributions

To assess EXB decoding accuracy, we examined the per-base mismatch rate between experimentally measured EXBs sequences and the designed barcode. We observed an average mismatch rate of ~0.1% across all sequenced bases in the EXB adapter region **(**Fig. 2a **)**. Among the possible 262,144 possible single-end EXBs, 100% were present in sequence data across all experiments. The experimentally observed EXB GC content also closely matched the theoretical distribution for all 6-mer barcode subunit combinations **(**Fig. 2b **)**, which suggested that the specific GC content of each EXB subunit did not significantly skew their representation.

### Molecular counting with EXBs

**Fig. 3:**
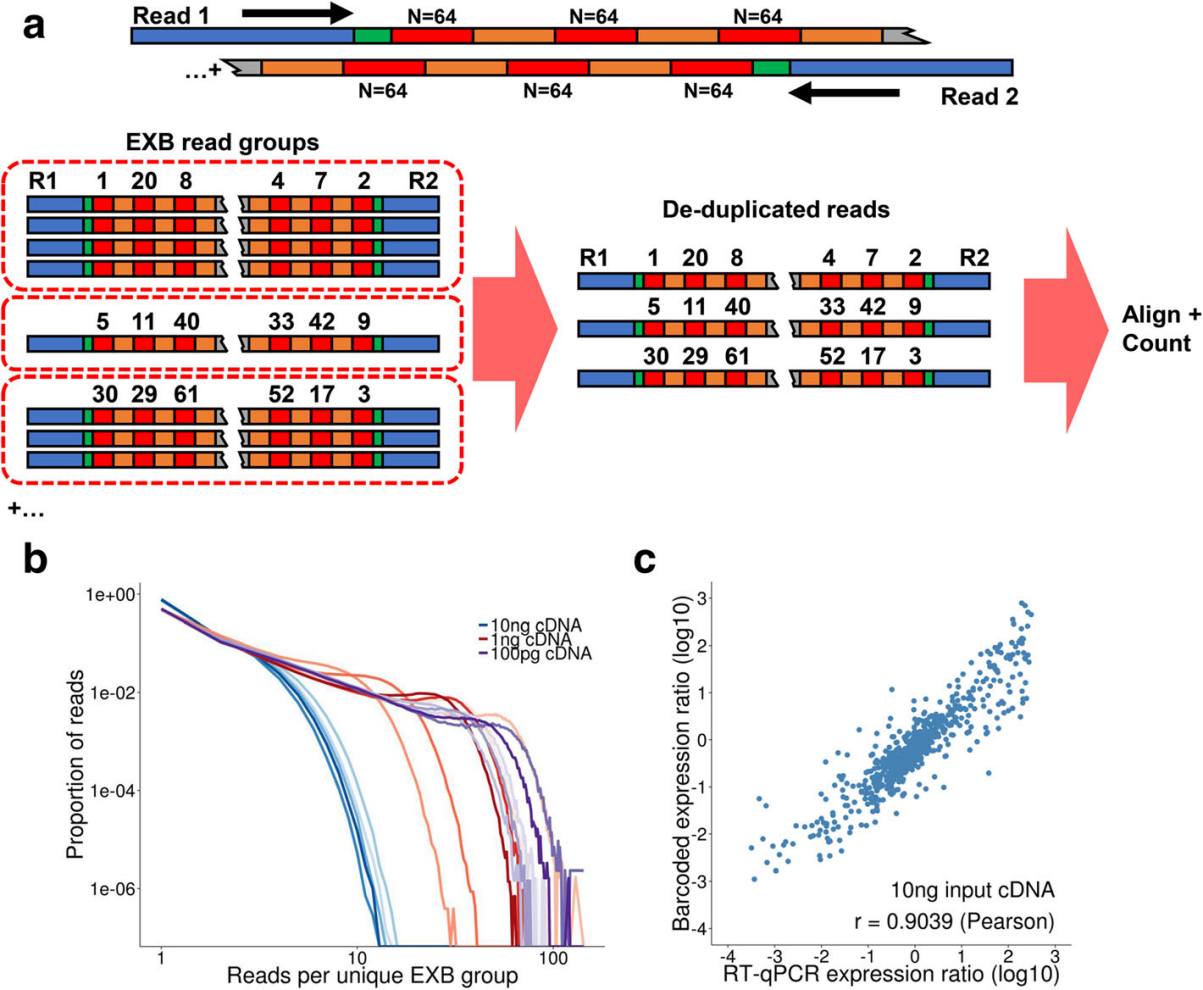
Transcriptome quantification with EXBs. **a** EXB processing workflow. Reads with the same paired-end barcode subunit sequences are group together into read groups. These read groups are then collapsed into consensus reads for downstream alignment and quantification. **b** EXB duplication rate. The number of aligning reads per paired-end EXB group is measured as a proportion of total reads. **c** Fold-change levels compared between previously determined MAQC RT-qPCR data and EXB-based expression levels for 10 ng input cDNA

We counted molecules using an alignment-free process. We grouped together all reads into EXB read groups, where all members possess the same decoded paired-end EXB sequence **(**Fig. 3a **)**. This approach relied on single-molecule tagging of sequenced fragments. We anticipated that the high diversity of unique EXBs would obviate the need for alignment-based positional indexing as required in molecular counting with random-mers [4–7, 9]. Assuming each cDNA fragment molecule would be labeled by a unique tag, this implied that all reads in an EXB read group should be ideally identical. To test this hypothesis, we measured the sequence similarity of the cDNA inserts per EXB read group, and observed that the median proportion of matching base pairs was 98.75 and 93.75% for read 1 and read 2 respectively. When considering sequencing error rates, this indicated that EXBs uniquely label single molecules.

We next considered EXBs based on the paired-end barcode structure. We observed ~105 million unique paired-end EXBs across sequencing data derived from 10 ng input cDNA **(**Additional file 1 **:** Table S3). The median EXB read group size was 1.0, and the barcode overlap rate was 0.397% across these samples. Decreasing cDNA amounts led to a corresponding increase in the number of reads in each EXB read group (Fig. 3b), with a mean of 76.4, 47.9, and 48.6% of EXB read groups being represented by only one read in the 10 ng, 1 ng, and 100 pg inputs respectively. To assess whether some paired-end EXBs are preferentially amplified during PCR, a Pearson correlation analysis of observed paired-end EXBs showed coefficients of less than 0.1 with 10 ng input cDNA (Additional file 1: Fig. S4). This result indicated a lack of biased amplification of specific EXB sequences.

### Gene expression analysis with EXBs

We assessed the performance of EXB-based transcriptome quantification. Expression counts of technical replicates at 10 ng cDNA had a Spearman correlation coefficient of over 0.99 across all genes **(**Additional file 1 **:** Figure S5), meaning that EXB tagging is highly reproducible across replicates. Smaller input amounts yielded lower Spearman correlation coefficients **(**Additional file 1 **:** Figure S6, Figure S7).

We next used the MAQC RT-qPCR dataset as a gene expression ground truth set to compare the gene expression quantification performance of EXBs, as qPCR is an orthogonal quantification platform and utilizes the fewest number of intermediate processing steps. We observed a Pearson correlation coefficient of ~0.90 when comparing qPCR fold-changes versus EXB-based expression at 10 ng **(**Fig. 3c, Additional file 3:Table S6, Additional file 4: Table S7). The correlation coefficient was 0.86 at 1 ng cDNA, and 0.72 at 100 pg input cDNA. As an independent validation with a custom qPCR gene panel (Methods), we observed an increased Pearson correlation of 0.85 for EXB-based expression on 100 pg of cDNA. This result implied that EXB-based tagging of cDNA produces gene expression results that are generally concordant with established datasets.

### Characterization of random molecular barcode errors with EXBs

We hypothesized that errors generated within random-mer molecular barcodes are difficult to detect. Furthermore, the extent of these errors has not yet been well characterized in other studies. While comparisons between assay technologies can be made, a more straightforward approach would be to include both molecular barcoding methods in tandem such that any sequenced fragment includes both types of tags.

**Fig. 4:**
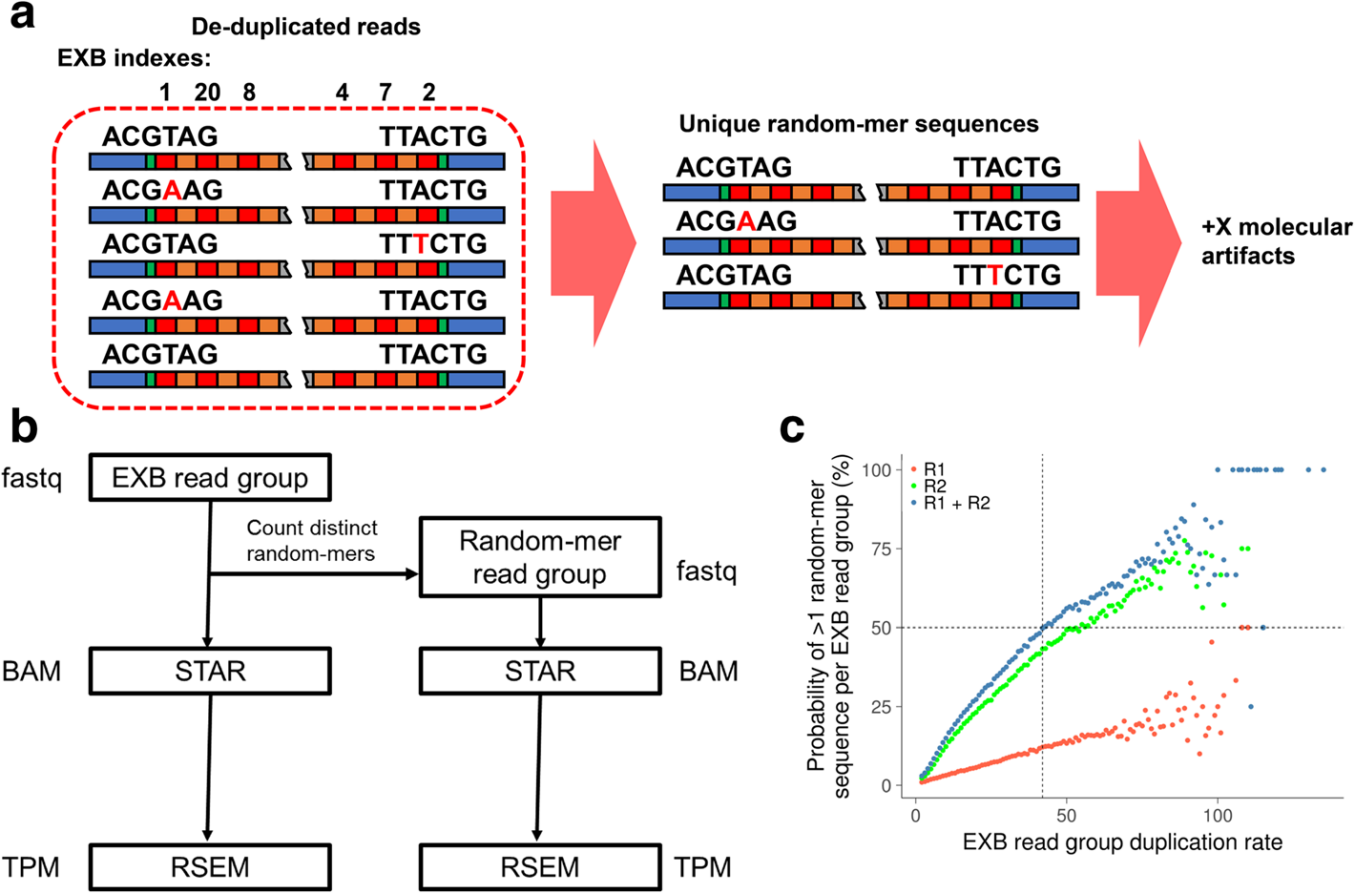
Random-mer molecular barcode characterization. **a** Processing workflow. The combined 12 bp random-mer sequences for each read (6 bp each for read 1 and read 2) in each EXB group are extracted. These represent new artefactual reads that are fed into downstream bioinformatic pipelines. **b** Comparison of EXB and random-mer workflows. After creating new artefactual reads, the two sets of reads are fed through identical pipelines for analysis. **c** Rate of mismatches found in random-mer sequences for each EXB group. Only EXB groups containing more than one read are considered. For each group of 12 bp random-mer sequences, the presence of any mismatches between them are considered and plotted as a function of the number of reads inside the EXB read group. The 12 bp random-mer sequence groups are also divided into 6 bp sequence groups corresponding to reads 1 and 2. One million reads from each library are pooled and grouped by paired-end EXB sequence

Here, we compared molecular counting results between EXBs and random-mer barcodes. The EXB structure included a 6 bp random sequence at the beginning of each read that enables direct comparison (Fig. 1a). As we above determined that an EXB read group likely consists of PCR duplicates of a single molecule, the random-mer barcode sequence should ideally be identical as well. To perform this analysis, we binned together reads by their corresponding EXB tag and counted the number of distinct random-mer sequences found in each group (Fig. 4a, Methods). Mismatching random-mer sequences in each EXB read group would correspond to new distinct reads. We then introduced new reads corresponding to every distinct random-mer species in an EXB read group which is then fed into identical downstream processing pipelines (Fig. 4b). The resulting gene expression counts was then compared to those processed solely with EXB tagging.

We binned the overall probability of finding non-unique random-mers by the number of reads found in an EXB read group to investigate the effects of PCR duplication. We observed that this probability increases with the level of EXB duplication (Fig. 4c). The probability of observing more than one unique random-mer sequence quickly saturated at a moderate duplication rate, which implied that this phenomenon may be widespread amongst molecular duplicates. At an EXB read group size of 42, there is on average a 50% probability of more than one distinct sequence detected in the random-mer barcode region. While we noted that the mismatch rate appears to be remarkably high, it is empirically calculated and specific to the context of this study; however, this effect was previously difficult to observe in other methods as they did not employ an orthogonal inline molecular barcode.

**Fig. 5:**
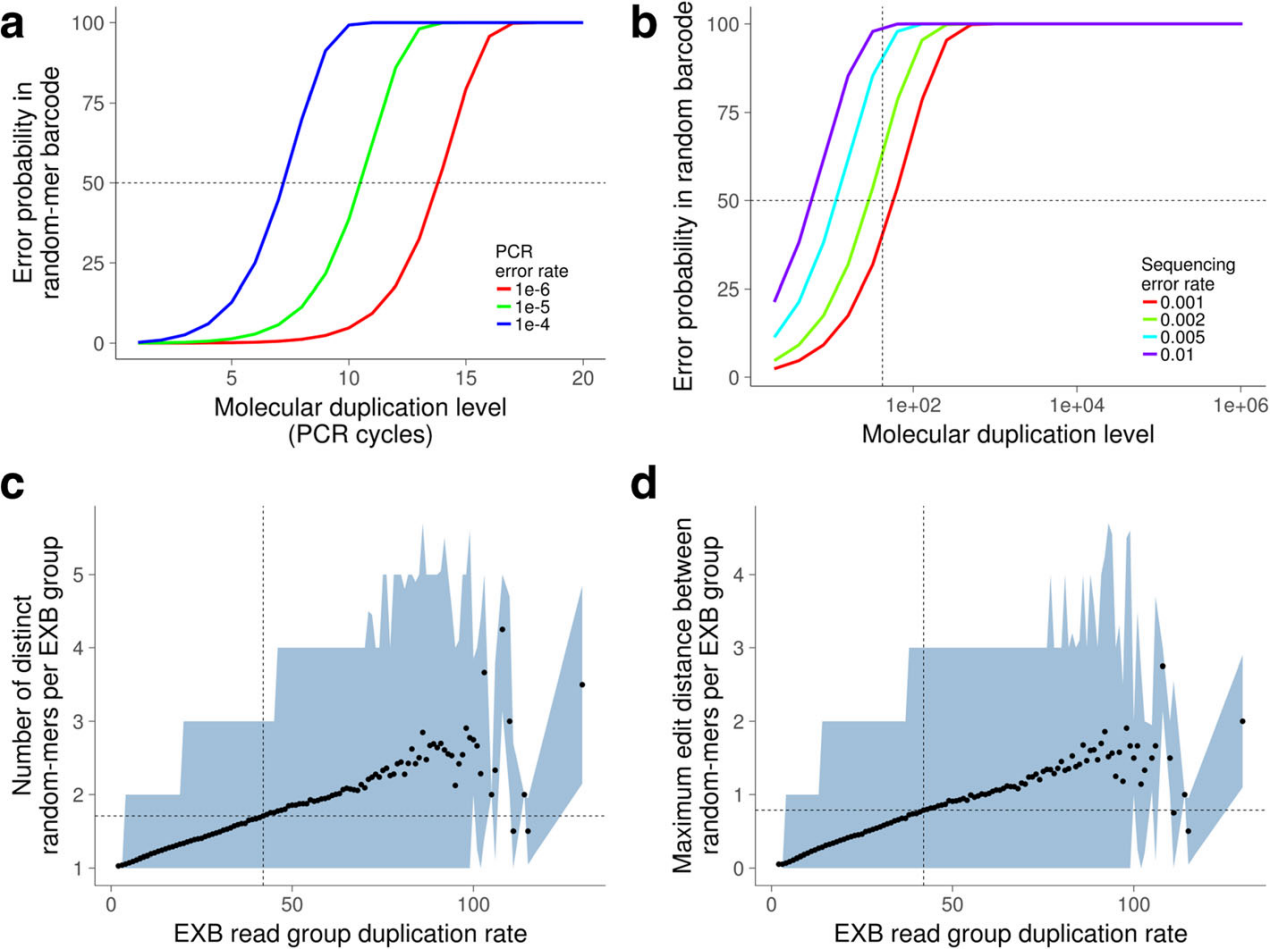
Modeling of random-mer barcode errors. Barcode error probabilities in the random-mer barcode sequence were modeled as being comprised of either **(a)** errors arising during PCR, or **(b)** errors arising during the sequencing assay. Error probabilities are calculated per initial library molecule. A simple model based on Poisson statistics was used; details are described in **Methods. c** Number of distinct random-mer sequences found in each EXB read group. The number of distinct random-mer sequences is measured as a function of the EXB read group size. Dots indicate the mean and the shaded area indicates the 5 and 95% quantiles. One million reads from each library are pooled and grouped by paired-end EXB sequence. **d** Edit distance between distinct random-mer sequences found in each EXB read group. The edit distance between distinct random-mer sequences is measured as the maximum edit distance between all random-mer sequences in a single EXB read group. Dots indicate the mean and the shaded area indicates the 5 and 95% quantiles

We hypothesized that errors in the random-mer molecular barcode sequence was due to the increasing chances of a polymerase-induced error occurring as the number of duplicate molecules increases. This can happen during library amplification and the sequencing assay. By using a simple probabilistic model based on Poisson statistics (Methods), we found that depending on the amount of amplification, PCR and sequencing errors both contribute substantially to the observed mismatches (Fig. 5a,b). When considering only sequencing errors, fitting the model against observed data (Fig. 4c) yielded an error rate of 0.136% (*p* < 0.001), which is in the range of Illumina sequencing error rates. The impact of this finding means that highly duplicated molecules are the most likely to contain barcode errors, and that this cannot be simply mitigated by using longer barcodes.

Experimentally, we observed that the number of distinct random-mer sequences found in an EXB read group also generally increased as a function of its duplication rate (Fig. 5c), which indicates an increasing problem of counting artefactual species. Moreover, the edit distance between random-mer molecular barcodes in an EXB read group also rapidly increased as a function of the duplication rate (Fig. 5d), supporting the modeling results. At EXB duplication rates where there was a 50% probability of non-unique random-mer sequences being detected, we observed a wide distribution in both the number of non-unique random-mer sequences as well as the maximum edit distance between them. For example, at an EXB duplication rate of 42, an edit distance filtering threshold of one would be insufficient to account for all of the possible errors in the associated random-mer barcode sequences. When taking these factors together, this leads to substantial difficulties in computational filtering of barcode errors.

### Assessing the impact of barcode errors on transcriptome analysis

We examined the impact of random-mer-based counting versus EXB-based counting. As genes were quantified in TPM units, which normalizes for the total number of molecular species, expression levels of one gene can be increased or decreased based on increased counts of another gene. Nonetheless, as errors in molecular counting will lead to false discovery of differentially expressed genes, we focused on genes that were discordant between methods.

**Fig. 6:**
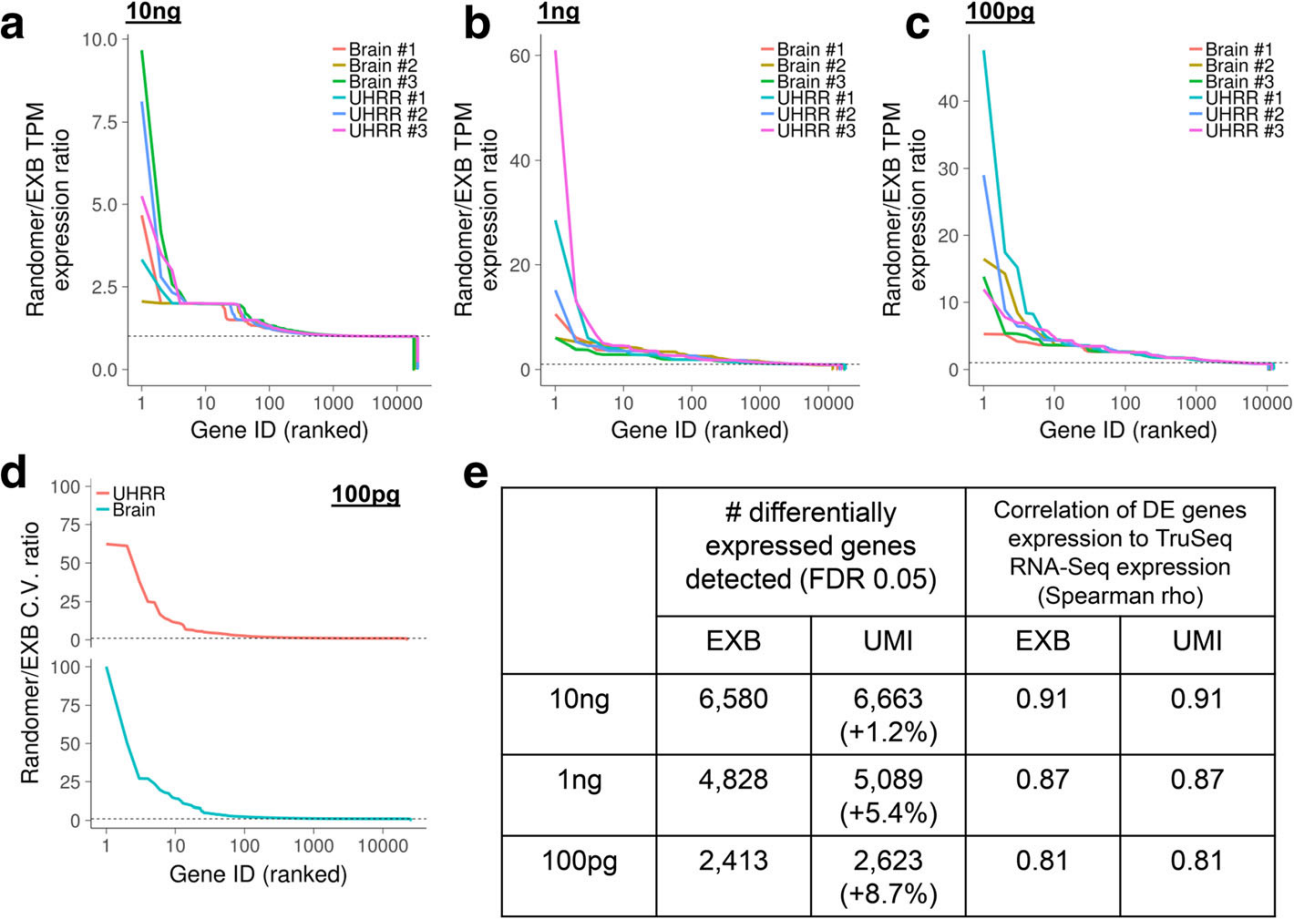
EXB-based and random-mer-based transcriptome analysis. The ratio between random-mer and EXB-based gene expression level is shown for **a** 10 ng, **b** 1 ng, and **c** 100 pg of input cDNA. The x-axis corresponds to the rank of the expression ratio, and is sorted independently for each sample. To emphasize the number of inflated genes, the x-axis is shown in log scale. **d** Technical variation between counting methods. The ratio between random-mer and EXB-based quantification coefficient of variation across technical replicates is shown for 100 pg input. For each sample type, the coefficient of variation ratios is sorted by decreasing order. **e** Impact of counting methods on differential expression analysis. The number of differentially expressed genes detected with an FDR threshold of 0.05 is shown for both random-mer and EXB-based quantification methods

We initially performed a Spearman correlation analysis on each library quantified with random-mer-based counting against previously determined qPCR-based expression levels. At first, we observed that the correlation coefficients were virtually identical to the EXB-based counting. However, we noted that the presence of outliers that did not substantially affect the correlation analysis due to the large number of genes counted. We then ranked the overall between-method ratio (ie. random-mer versus EXB-based counting) in gene expression levels for each replicate and each sample type. As a result, a substantial number of genes have inflated expression levels when not accounting for errors in the random-mer barcode sequence (Fig. 6a–c). In the 100 pg input cDNA samples, approximately ~10% of genes with non-zero expression had a fold-change increase of greater than 1.5, indicating a general transcriptome-wide increase in artefactual counts when using random-mer-based molecular counting. In general, artefactual counts were not biased towards particular transcript abundances for 100 pg input cDNA **(**Additional file 1 **:** Figure S8), with an average Pearson correlation coefficient of −0.007 ± 0.001 (s.e.m., *p* > 0.05) between the inflated random-mer-to-EXB counts ratio and the actual EXB expression. We noted that the effect is substantially less pronounced at 10 ng of input cDNA, supporting that this phenomenon is driven by artefactual molecular counting when starting with low input amounts.

We hypothesized that increased counts from undetected errors in random-mer-based counting would lead to inflated variance across replicates. Although less than 100 genes in the 10 ng cDNA input assays showed large discrepancies between counting methods, approximately 2000 genes in the 100 pg input (Fig. 6d) corresponding to over 10% of expressed genes across both sample types displayed higher levels of technical variance when counting using random-mers versus EXBs. This unpredictable confounding factor would subsequently impede downstream gene expression analysis. To assess the impact on differential gene expression analysis, we used the quantified TPM expression values under both EXB and random-mer pipelines to search for differentially expressed genes (Methods, Fig. 6e). Even with a generally good Spearman correlation with external RNA-Seq datasets (Fig. 6e), we observed a generally increased number of differentially expressed genes when counting random-mer molecular barcodes. The effect was reduced at 10 ng input cDNA, but rises to almost 10% at 100 pg of input. Interestingly, when using an edit distance of 1 for random-mer barcode error correction, the number of differentially expressed genes was less than the EXB result. We observed 6440, 4760, and 2376 differentially expressed genes for 10 ng, 1 ng, and 100 pg input cDNA respectively, which supports previous studies indicating that barcode correction algorithms simply involving edit distance may not recapitulate the complex phenomena of molecular barcode errors [16]. We further spot-checked several genes to visualize the aggregate effects of false gene expression counts and increased technical variance **(**Additional file 1: Figure S9). This led to the conclusion that as the input quantity decreases, the errors derived from incorrect counting of random-mer molecular barcodes became increasingly pronounced.

## Discussion

In this proof-of-concept study, we described the novel use of error correcting sequences to generate exponentially large amounts of molecular barcodes. Because the diversity of EXBs scale with length of barcode subunit and to the number of repeats, the method could be extended to even greater diversities as sequencing read lengths and capacities develop over time. For example, expanding the number of subunits from six to eight results in approximately peta-scale diversity. The assembly protocol for combining barcode subunits is flexible, and can be modified for any study and sequencing platform of interest.

The scaffold structure required to synthesize the EXB adapter necessitates the use of longer reads and adds to the total sequencing reagent cost. In this study, we utilized a 2 × 151 sequencing scheme, which is more expensive than conventional 2 × 75 or even 1 × 75 sequencing assays used in many transcriptome studies. Random-mer barcodes often require over-sequencing such that many molecules are represented more than once. This substantially reduced the number of reads that are informative for molecular counting. EXBs, by virtue of error correction, can be counted as a bona fide molecule even when represented as a singleton. Therefore, EXBs would require less total reads and thus would reduce the sequencing cost. The exact balance between between read length and sequencing depth would be dependent on the specific details of any assay; however, we note that sequencing lengths continue to increase among next-generation sequencing technologies.

The results from this proof-of-concept study showed several clear paths for future methodological improvements. The applicability of EXBs relies on its adaptation towards other experimental assays. Although random-mer barcodes, commonly in the form of an ssDNA poly-T-tailed primer for reverse transcription, are simple and inexpensive to synthesize, the inherent presence of difficult-to-correct errors make them risky to employ and motivates the adoption of alternative barcoding methods. Overall, we envision that numerous improvements of EXB-style molecular barcodes can be explored in future studies, whereby EXBs can be incorporated into an ssDNA-based adapter or the scaffold lengths can be reduced to lower sequencing costs.

Because EXBs can reliably label single molecules and contain an inline random nucleotide sequence, we were able to for the first time investigate errors that arise in random-mer molecular barcodes. We surprisingly observed a high number of EXB read groups that contained more than one distinct random-mer sequence. This is nonetheless consistent with our basic probabilistic models of errors arising in both PCR and sequencing. While strategies such as random-mer sequence similarity grouping or abundance filtering can be used to address this phenomenon, the fundamentally random nature of random-mer molecular barcodes ultimately leads to uncertainty in any correction scheme.

We note again that the observed error rates in this study are context-specific and will change from study-to-study due to technical differences in library preparation and sequence data quality. For example, the type of polymerase used and the specific amplification method leads to different error profiles. Furthermore, the structural properties of a transposable adapter may display substantially different tagging and sequencing performance versus a single-stranded molecular barcoded oligonucleotide that is commonly used for tagging cDNA. Nonetheless, random-mer barcode errors are difficult to detect without an inline rational barcoding strategy, and are near impossible to correct when edit distances between error-containing molecular barcodes are substantial. Experimentally, we observed that the impact of errors in random-mer molecular barcodes becomes considerably worse as the input amount decreases. This would have profound impacts in single-cell RNA-Seq studies, and will be a topic of further study.

We anticipate that EXBs would also be useful for sequencing platforms with substantially higher substitution/indel sequencing error rates such as on Pacific Biosciences or Oxford Nanopore sequencers. Correction of indels is not possible with decoder matrix-based error correction, but other methods such as Levenshtein distance metrics can be used to match to the closest barcode. Furthermore, the total throughput of these sequencers still needs to be increased in order for molecular counting to become feasible. As the capacity of these sequencing technologies improve, EXBs would in the future be an attractive option for molecular barcoding.

We have described the use of EXBs to robustly identify and count single molecules for transcriptome studies. In contrast to random-mer molecular barcoding, EXBs robust to PCR and sequencing errors that can generate false positive counts. In comparison to other molecular barcoding methods (Additional file 1: Table S8), EXBs are the first type of molecular barcode that enables high diversity molecular tagging with rational barcodes via transposase-based insertion. Overall, we anticipate that EXBs will be particularly useful for the quantification of single molecules that are found in miniscule abundances.

## Conclusion

In this study, we have demonstrated the first use of transposable molecular barcodes for transcriptome quantification. By applying the method to cDNA standards, we characterized the method’s performance. Furthermore, we also demonstrated the first systematic detection and characterization of errors that may occur in random-mer molecular barcodes. We discovered that such errors are extensive, which may have large impacts for studies utilizing miniscule amounts of transcriptome material.

## Methods

### Molecular barcode design

EXBs are comprised of combinatorially expanded set of 6 bp barcodes along scaffolds. To generate each barcode, we used an optimal linear generator matrix in quaternary space where the bases A, C, G and T correspond to the integers 0, 1, 2 and 3. We performed matrix multiplication between all possible 3-mer sequences (*N* = 64). The output is a set of 64 6 bp barcode sequences where the last 3 bp of each barcode corresponds to a linear combination of the first 3 bp as specified by the generator matrix **(**Additional file 1: Table S1). The specific generator matrix is known as the Hexacode [24] and has been characterized as an optimal generator matrix where the edit (substitution) distance between possible barcodes is maximized. The edit distance between these barcodes can be determined by pairwise comparison (Fig. 1b, Additional file 1: Figure S1).

### Molecular barcode synthesis and assembly

To combinatorially expand these barcodes, we used a modified extension-ligation approach [25]. Subunits containing a single 6 bp barcode are annealed along four common scaffold regions A, B, C and the Illumina sequencing adapter (Fig. 1a, Additional file 1: Figure S2a). We synthesized 192 oligonucleotides via column synthesis (Sigma-Aldrich) corresponding to each of the possible 6 bp barcodes flanked by the Illumina adapter and A; A and B; and B and C (Additional file 2: Table S2). Barcodes flanked by the Illumina adapter and A also contain a random-mer sequence of 6 bp. We also synthesized two additional oligonucleotides corresponding to the reverse complement of the Tn5 recognition sequence and the reverse complement of the Illumina multiplexed Read 2 sequence (Additional file 2: Table S2). All oligonucleotides were normalized to 100 μM in 10 mM Tris-HCl, pH 8.0 buffer. Afterward, oligonucleotides of the same subunit group (ie. those with common flanking scaffolds) were pooled together.

To perform the extension-ligation reaction, we combined 9 μl of each oligonucleotide pool corresponding to the three-subunit groups with 9 μl of each common oligonucleotide into an 8-strip PCR tube. We then heat-denatured this mixture by incubation in a thermocycler for 5 min at 95C and quickly placing the tube on ice. To this mixture we further added 50 μl of 2× Quick Ligase Buffer (New England Biolabs) and 5 μl of Optikinase (Affymetrix). After pipet mixing we incubated this tube at 37C overnight in a thermocycler.

After incubation, we heat denatured the oligonucleotide mixture at 95C for 5 min and then held forever at 50C with a ramp of 1C/min. During the ramping process, we combined in a separate tube 20 μl of 5× assembly reaction buffer [25], 40 μl Betaine (Sigma-Aldrich), 2.5 μl Phusion polymerase (New England Biolabs), 4 μl high concentration Ampligase (Epicentre), 12.5 μl 40% PEG 8000 (Sigma-Aldrich), 1 μl thermostable inorganic pyrophosphatase (New England Biolabs) and 20 μl molecular grade water. Once the thermocycler reached 50C, we pipetted the enzyme mixture into the oligonucleotide mixture while leaving the PCR strip tube at 50C. After gentle pipet mixing, we split the final mixture into two by pipetting 100 μl into the adjacent well on the strip tube, sealed the strip tube with a new lid, closed the thermocycler, and incubated for another 48 h at 50C (Additional file 1: Figure S2a).

The contents of the two wells were pipetted into a 1.7 ml microcentrifuge tube. 600 μl of Ampure XP beads (Beckman Coulter) were added to the mixture and mixed well. Immediately afterward we also added 200 μl of molecular grade isopropanol (Sigma-Aldrich). This mixture was incubated for 15 min before placing on a microcentrifuge tube-sized magnetic stand (Promega) for 10 min. The collected beads were rinsed twice with 90% ethanol, air-dried for 10 min, and then resuspended in 100 μl 10 mM Tris-HCl, pH 8.0 buffer. The assembled adapter is then quantified by absorbance with a Nanodrop instrument.

### Transposase expression and purification

The plasmid psfTn5-c006 was a kind gift from Sten Linnarsson’s group. The plasmid is a modification of an existing protocol to express Tn5 transposase [26], and encodes an N-terminal His-tagged hyperactive Tn5 transposase under T7-controlled expression inside a pNIC-Bsa4 vector. This plasmid was transformed into ArcticExpress competent cells (Agilent) with standard protocols and plated under kanamycin selection. A single colony was picked and inoculated into 50 ml of auto-inducing Magic Medium (Life Technologies) with 50 μg/ml kanamycin selection. The culture was incubated at room temperature for 24 h in a 180 rpm shaking platform at room temperature, and then placed in a cold room for 4 h. The culture was poured into two 50 ml conical tubes, centrifuged at 4000 g, and stored as pellets at -80C.

To lyse the pellets, 10 ml of lysis buffer (6 ml B-PER Complete, 2.5 ml BioStab enzyme stabilizer (Sigma-Aldrich), 1 ml 5 M NaCl, 2 μl 500 mM EDTA, 1 ml glycerol, 50 μl 2 M imidazole, and 50 μl Protease Inhibitor Cocktail (Promega)) was added. The pellet was gently resuspended with a 10 ml serological pipet and the mixture was rotated for an hour at 4C. The crude lysate was centrifuged at 4000 g for 20 min, and then was passed through a prepacked and equilibrated HisPur Ni-NTA column at 700 g. The column was washed three times with wash buffer (100 mM Tris-HCl 8.5, 250 mM NaCl, 0.1 mM EDTA, 10% glycerol, 25 mM imidazole) before elution with 3 ml eluting buffer (50 mM Tris-HCl, pH 7.5, 100 mM NaCl, 0.1 mM EDTA, 1X BioStab enzyme stabilizer, 500 mM imidazole, 25% *v*/v glycerol). The enzyme is quantified by a Nanodrop instrument, after which Triton X100 (Promega) and DTT (Sigma-Alrich) is added to a final concentration of 0.1% and 1 mM respectively. The enzyme is normalized to 4 μg/ml and stored at -20C.

Purified transposases are loaded with the molecular barcode adapter by combining 1.5 μl of transposase enzyme, 25 pmol of adapter, and bringing up to 20 μl with a solution of 50% glycerol and 1X BioStab enzyme stabilizer. The mixture is then incubated at room temperature for 1 hour at room temperature before placing on ice or storage at -20C.

### Sample library preparation and sequencing

Purified brain and human reference RNA (Life Technologies and Agilent) used for the MAQC/SEQC projects [23] were used for this study. Full-length double-stranded cDNA was generated from 5 μg of starting material using the Maxima H- double-stranded cDNA synthesis kit (Life Technologies) under standard protocols. This stock was diluted down to 10 ng/μl, 1 ng/μl, and 100 pg/μl immediately before library generation.

We performed transposition of full-length DNA by combining on ice 1 μl of diluted cDNA stock with 1 μl transposase in 1× TD buffer (10 mM TAPS, pH 8.5, 5 mM MgCl2, 5% v/v DMF) and incubating at 55C for 15 min before returning to ice. 1 μl of 10% sodium dodecyl sulfate (SDS) detergent (Sigma-Aldrich) was added, and the mixture was incubated for a further 15 min at 55C. The mixture was then purified with 1.8X Ampure XP beads under standard manufacturer protocols.

To fill gaps generated by Tn5 transposase, the magnetic beads are resuspended in 20 μl of 1X Ampligase buffer (Epicentre), 0.25 mM dNTP, 0.75 U T4 DNA Polymerase (New England Biolabs), 2.5 U *E. coli* DNA Ligase (New England Biolabs), and 5 μg/μl T4 Gene 32 Protein (New England Biolabs). T4 gene product 32 inhibits single-stranded exonuclease activity of T4 DNA polymerase but allows for polymerase activity to proceed [27]. The mixture is then incubated at 25C for 1 hour followed by 65C for 20 min. To this mixture, 30 μl of molecular grade water and 50 μl of SPRI solution (13% PEG, 2.5 M NaCl, 0.05% Tween-20) is added, followed by bead magnetization, aspiration, and washing with 80% ethanol. The beads are then resuspended with PCR amplification mix (1X KAPA HiFi HotStart ReadyMix (Kapa Biosystems), 1X BioStab PCR Optimizer (Sigma-Aldrich), 500 nM universal PCR primer, 500 nM sample index primer, 0.5 U Thermostable Inorganic Pyrophosphatase (New England Biolabs)) and amplified with the following protocol: 45 s at 98C, *C* cycles of 15 s at 98C, 30 s at 65C, 90 s at 72C, and 5 min at 72C. *C* corresponds to 18 cycles, 21 cycles, and 24 cycles for 10 ng, 1 ng, and 100 pg input cDNA respectively. The amplified libraries are purified with 1.8X Ampure XP beads with standard protocols. 1 μg of PCR product is loaded into one lane of a 1.5% Pippin Prep (Sage Sciences) cartridge and size selected to 500-1000 bp on the Pippin Prep instrument (Sage Sciences). The size-selected fragments are purified again with 0.65X Ampure XP beads with standard protocols, and are then quantified by qPCR (Kapa Biosystems) using Illumina P5 and P7 primers and 600 bp insert size. Libraries are then pooled and normalized to 2 nM before sequencing on a HiSeq 2500 under Rapid Run v2 mode with ~15% PhiX. To prevent mispriming by sequencing primers complementary to the Tn5 recognition sequence used by Nextera-based libraries, we loaded the TruSeq R1 (5′- ACACTCTTTCCCTACACGACGCTCTTCCGATCT) and R2 oligonucleotides (5′- GTGACTGGAGTTCAGACGTGTGCTCTTCCGATCT and 5′- CGGTCTCGGCATTCCTGCTGAACCGCTCTTCCGATCT) as custom sequencing primers.

### Barcode processing

After generation of FASTQ files with bcl2fastq (Illumina), barcodes are processed using a custom multithreaded Python (v2.7) scripts (https://github.com/billytcl/EXB). The six 6 bp segments corresponding to barcode subunits from both reads (three 6 bp segments from read 1, and three 6 bp segments from read 2) are individually extracted and decoded using a decoder matrix (Additional file 1: Figure S3). These six-number tuples correspond to the indexes of a hash table in which all reads are grouped.

Perfectly matching 6 bp subunits, when multiplied into the decoder matrix, result in a vector of zeros. Mismatches in the 6 bp sequence are detected as a non-zero vector in the decoder matrix output, known as a syndrome. Syndromes correspond to a set of possible error patterns, that when reversed allow for the measured error-containing barcode to match those that are generated by the generator matrix (Additional file 1: Figure S3). We utilized the principle of parsimony and selected for error patterns of the lowest edit distance; for example, edit distance patterns requiring only one substitution are selected over those requiring two. We set aside reads containing multiple error patterns with the same edit distance; at the end of decoding analysis we checked from the final set of decoded barcodes for the presence of a barcode matching every possible result of error correction. Given the observation of low EXB overlap between distinct DNA fragments, we searched for matches between an error-prone EXB and one from the observed pool. If none of the possible EXBs were previously observed in the sample, then the error-prone barcode was identified as a unique molecule to be used for counting.

For each group of reads, a consensus is formed based on majority counts of the base call. If a majority cannot be distinguished (eg. two bases of equal abundance), the final consensus base is output as *N. consensus* reads are output as interleaved paired-end FASTA files with no quality scores, and then trimmed using cutadapt [28] using the parameter -b CTGTCTCTTATACACATCT.

To compare our molecular barcoding strategy to those utilizing random barcodes, we included a 6 bp random nucleotide sequence immediately after the end of the read 1 and read 2 (12 bp in total) sequencing primer sites (Fig. 1a). After grouping of our molecular barcodes, we extracted only those which had a duplication rate of greater than 1. From these reads we measured the rate at which any of the 12 bp sequences were discordant amongst reads belonging to a single EXB read group.

### RNA-Seq alignment and quantification of MAQC transcriptomes

Consensus sequencing reads are aligned using STAR [29] (v2.4.2) by de-interleaving the output FASTA file from above. We used the GRCh37.75 ENSEMBL release with the following options:

--outSAMtype BAM SortedByCoordinate --quantMode TranscriptomeSAM GeneCounts --outSAMunmapped Within --outFilterType BySJout --outSAMattributes NH HI AS NM MD --outFilterMultimapNmax 20 --alignSJoverhangMin 8 --alignSJDBoverhangMin 1 --sjdbScore 1 --outFilterMismatchNmax 999 --alignIntronMin 20 --alignIntronMax 1000000 --alignMatesGapMax 1000000 --outFilterMatchNminOverLread 0 --outFilterScoreMinOverLread 0. This increases the sensitivity of the alignment while still providing excellent concordance to MAQC expression standards. The parameters are also are similar to the parameters used by ENCODE and built into RSEM (https://github.com/deweylab/RSEM/blob/master/rsem-calculate-expression) [30].

The resultant aligned reads are then quantified with RSEM [30, 31] (v1.2.22) using the following options:

--bam --estimate-rspd --no-bam-output --seed 12345 --paired-end --no-qualities.

We use the gene level transcript abundance estimates for downstream analyses.

We used the tool EBSeq [32] to detect differentially expressed genes. It is built into the RSEM (v1.2.22) as an integrated pipeline. We used a stringency false discovery rate (FDR) cutoff of 0.05.

### Comparison of EXB-based and random-mer processing

To compare between the two methods, the 12 bp random-mer sequence associated with each read in each EXB-read group (6 bp for each read) is tabulated to determine the number of unique random-mer sequences. Similar to EXB-based processing, a consensus FASTA sequence identical to the EXB-based output is generated, but with an extra read for every distinct random-mer sequence observed. This corresponds to an inflation in the total number of molecules detected. This output is then fed into identical processing workflows to determine alignment, gene expression levels, and differential expression.

To compare differential expression with an external RNA-Seq dataset, we downloaded the public dataset corresponding to TruSeq stranded mRNA libraries performed on the MAQC samples and sequenced on the HiSeq 2500 as provided on Illumina Basespace. Identical alignment and expression quantification workflows were performed.

### Modeling of errors in random-mer sequences

We used a simple model to investigate the rate at which errors arise in random-mer molecular barcodes. We hypothesize that there are two major sources of error: during PCR, and during the sequencing assay.

Errors during the PCR process can be modeled by a simple Poisson model. Given a PCR error rate *e*, barcode length *l*, and number of duplicated molecules *m*, the average error rate for a single starting template molecule would be *λ = elm*. For sufficiently small *λ*, we can use a Poisson distribution and determine the probability of no error occurring for a single starting template molecule as *p*_0_ = exp(−*λ*) = exp(−*elm*). Additionally, we have to consider that errors during PCR have multiple chances to occur at each amplification cycle; therefore, the probability of no errors over the entire PCR process for a single starting template molecule is approximately ${p}_0(c)=\prod \limits_1^c\exp \left(-{el}^{\ast }{2}^c\right)=\exp \left(-{el}^{\ast }2\left(-1+{2}^c\right)\right)$ where *m = 2* ^*c*^. The probability of a single starting template molecule receiving an error during the PCR process is thus *p*_*error*_(*c*) = 1 − *p*_0_(*c*).

Errors that occur during the sequencing process can also be similarly modeled. As above, given a sequencing error rate *e*, barcode length *l*, and number of duplicated molecules *m*, the average error rate for a single starting template molecule would be *λ = elm*. For sufficiently small *λ*, we can use a Poisson distribution and determine the probability of no error occurring for a single starting template molecule as *p*_0_ = exp(−*λ*) = exp(−*elm*). Here, we do not need to consider the cumulative effects of each PCR cycle, and thus the random-mer error probability of a single starting template molecule that has been duplicated to *m* copies is *p*_*error*_(*c*) = 1 − exp(*elm*).

### Custom gene panel qPCR

We loaded 100 pg of cDNA from either the brain or human MAQC standard into each well of a 384-well SAB cell lineage identification panel (Bio-Rad)**.** Each well, in addition to loaded cDNA, contained 1X SsoAdvanced Universal SYBR Green Supermix (Bio-Rad) in a total of 10ul. Each sample type was run in quadruplicate to occupy an entire 384-well plate. We performed qPCR on an Applied Biosystems 7900HT qPCR system under the following conditions: 95C for 2 min, then 30 cycles of 95C for 5 s and 60C for 30 s. Ct values were automatically generated with standard software settings. We filtered for reaction dropout by requiring all replicates for a target gene to have a non-zero and finite Ct. We then averaged across replicates for each gene and generated fold-changes by dividing one sample type by the other.

## Additional files

Additional file 1: Figure S1. Pairwise comparison of EXB barcode subunits. **Figure S2.** Enzymatic construction of EXBs. **Figure S3.** EXB analysis workflow. **Figure S4.** Barcode correlation between paired-end EXB groups. **Figure S5.** Gene expression correlation between EXB-labelled transcriptome data at 10 ng input. **Figure S6.** Gene expression correlation between EXB-labelled transcriptome data at 1 ng input. **Figure S7.** Gene expression correlation between EXB-labelled transcriptome data at 100 pg input. **Figure S8.** Artefactual effects of random-mer-based expression analysis as a function of EXB expression level. **Figure S9.** Technical variation between counting methods. **Table S1.** List of barcodes generated by linear coding matrix. **Table S3.** Sequencing statistics for 10 ng input cDNA assays. **Table S4.** Sequencing statistics for 1 ng input cDNA assays. **Table S5.** Sequencing statistics for 100 pg input cDNA assays. **Table S8.** Comparison to other molecular barcoding sequencing methods. (PDF 7740 kb)

Additional file 2: Table S2. List of oligonucleotides. (TXT 11 kb)

Additional file 3: Table S6. Comparison of MAQC qPCR and EXB-based quantification methods. (TXT 90 kb)

Additional file 4: Table S7. Comparison of MAQC qPCR fold-change and EXB-based fold-change quantification methods. (TXT 61 kb)

## Abbreviations

EXB: Exponentially-expanded barcodes
MAQC: Microarray Quality Control
