# Supplementary material for: Single molecule counting and assessment of random molecular tagging errors with transposable giga-scale error-correcting barcodes
Source: BMC Genomics. 2017 Sep 21;18:745. doi: 10.1186/s12864-017-4141-4 (PMC5609065; doi:10.1186/s12864-017-4141-4)
Supplement: Supplementary file 1 — Pairwise comparison of EXB barcode subunits. Figure S2. Enzymatic construction of EXBs. Figure S3. EXB analysis workflow. Figure S4. Barcode correlation between paired-end EXB groups. Figure S5. Gene expression correlation between EXB-labelled transcriptome data at 10 ng input. Figure S6. Gene expression correlation between EXB-labelled transcriptome data at 1 ng input. Figure S7. Gene expression correlation between EXB-labelled transcriptome data at 100 pg input. Figure S8. Artefactual effects of random-mer-based expression analysis as a function of EXB expression level. Figure S9. Technical variation between counting methods. Table S1. List of barcodes generated by linear coding matrix. Table S3. Sequencing statistics for 10 ng input cDNA assays. Table S4. Sequencing statistics for 1 ng input cDNA assays. Table S5. Sequencing statistics for 100 pg input cDNA assays. Table S8. Comparison to other molecular barcoding sequencing methods. (PDF 7740 kb) [file 12864_2017_4141_MOESM1_ESM.pdf]

SUPPLEMENTARY INFORMATION

Single molecule counting and assessment of random molecular tagging errors with transposable giga-scale error-correcting barcodes

AUTHORS

Billy T. Lau[1], Hanlee Ji[1,2]

[1]Stanford Genome Technology Center, Stanford University, Palo Alto, CA, 94304, United States

[2]Division of Oncology, Department of Medicine, Stanford University School of Medicine, Stanford, CA, 94305, United States

TABLE OF CONTENTS

Supplementary Figure S1. Pairwise comparison of EXB barcode subunits.

Supplementary Figure S2. Enzymatic construction of EXBs.

Supplementary Figure S3. EXB analysis workflow.

Supplementary Figure S4. Barcode correlation between paired-end EXB groups.

Supplementary Figure S5. Gene expression correlation between EXB-labelled transcriptome data at 10ng input.

Supplementary Figure S6. Gene expression correlation between EXB-labelled transcriptome data at 1ng input.

Supplementary Figure S7 Gene expression correlation between EXB-labelled transcriptome data at 100pg input.

Supplementary Figure S8. Artifactual effects of random-mer-based expression analysis as a function of EXB expression level.

Supplementary Figure S9. Technical variation between counting methods.

Supplementary Table S1. List of barcodes generated by linear coding matrix.

Supplementary Table S2. List of oligonucleotides. As separate file.

Supplementary Table S3. Sequencing statistics for 10ng input cDNA assays.

Supplementary Table S4. Sequencing statistics for 1ng input cDNA assays.

Supplementary Table S5. Sequencing statistics for 100pg input cDNA assays.

Supplementary Table S6. Comparison of MAQC qPCR and EXB-based quantification methods. As separate file.

Supplementary Table S7. Comparison of MAQC qPCR fold-change and EXB-based fold-change quantification methods. As separate file.

Supplementary Table S8. Comparison to other molecular barcoding sequencing methods.

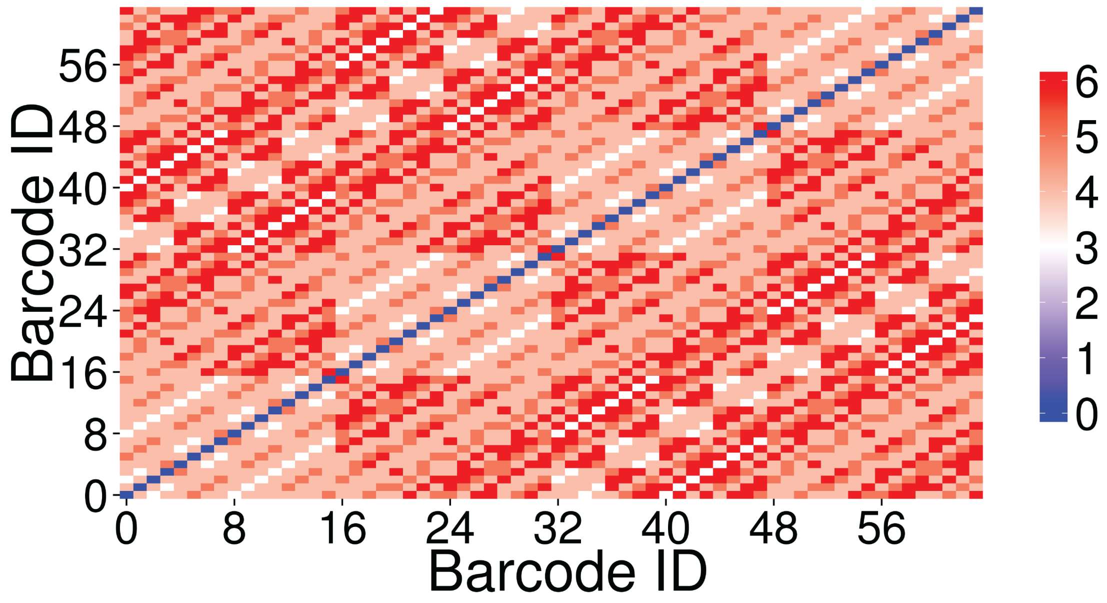

Supplementary Figure S1. Pairwise comparison of EXB barcode subunits. The edit distance (substitution distance) between each of the 64 6bp EXB subunits were compared against each other. The midpoint of the heatmap is set at an edit distance of 3, evidently indicating that each EXB subunit is substantially different from each other by over 50% of the subunit length.

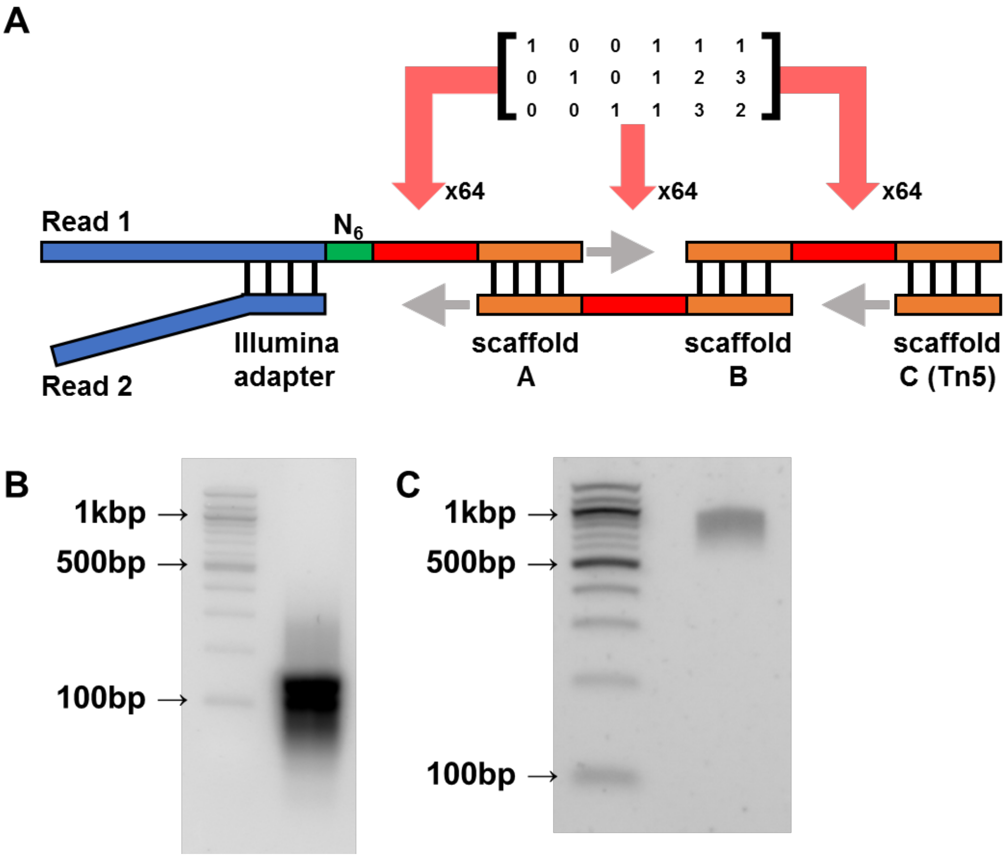

Supplementary Figure S2. Enzymatic construction of EXBs. (A) EXBs are assembled in an extension-ligation reaction. Two common oligonucleotides are annealed together with 192 oligonucleotides divided into 3 groups of EXB subunits, each of which contain 64 members. After annealing, an extension-ligation reaction is performed to generate an adapter on which to load onto Tn5 transposase. A forked adapter enables the construction of paired-end libraries during transposition. **(B)** Gel electrophoresis image of assembled adapter structure. The assembled size is ~130bp; however the ssDNA portion of the forked adapter causes smearing in the gel. Lane 1 is Quick-Load 100bp DNA ladder (New England Biolabs). 100bp, 500bp, and 1kbp reference DNA sizes are annotated. **(C)** Gel electrophoresis image of a final library product after PCR and size selection. Libraries were processed as described in **Methods**, where they are PCR amplified and size-selected on a Pippin Prep instrument. Lane 1 is Quick-Lpoad 100bp DNA ladder (New England Biolabs). 100bp, 500bp, and 1kbp reference DNA sizes are annotated.

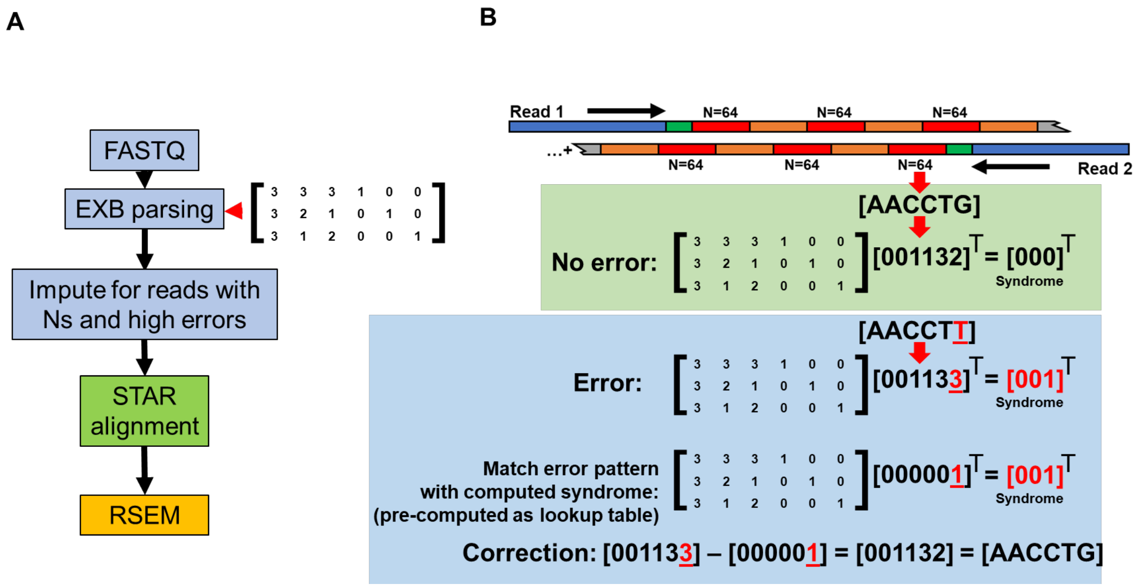

Supplementary Figure S3. EXB analysis workflow. (A) Pipeline overview. FASTQ files are first parsed for EXBs by using a linear decoder matrix. A linear decoder matrix assists in matching sequenced EXBs to those that were designed by detecting and correcting for experimental sequence errors. EXBs are then grouped together, after which a consensus sequence is generated for alignment. Sequences containing Ns and a high number of errors in the EXB as determined by the decoder matrix have many possible matches to the designed set, and are set aside until the end of the decoding process. A likely match can then be imputed by searching in the observed pool of highly confident decoded EXBs. After decoding, the consensus sequences are then aligned and quantified with STAR and RSEM. **(B)** Error correction example. 6bp EXB subunits are read along the sequencing read and are decoded by the 6x3 decoder matrix as shown. The output is a syndrome, which is an indication of whether there is no error (green box), or whether an error is detected (blue box). Upon detection of an error, the syndrome is matched to a precomputed error pattern. This error pattern can be subtracted from the measured sequence to achieve an error-corrected EXB subunit.

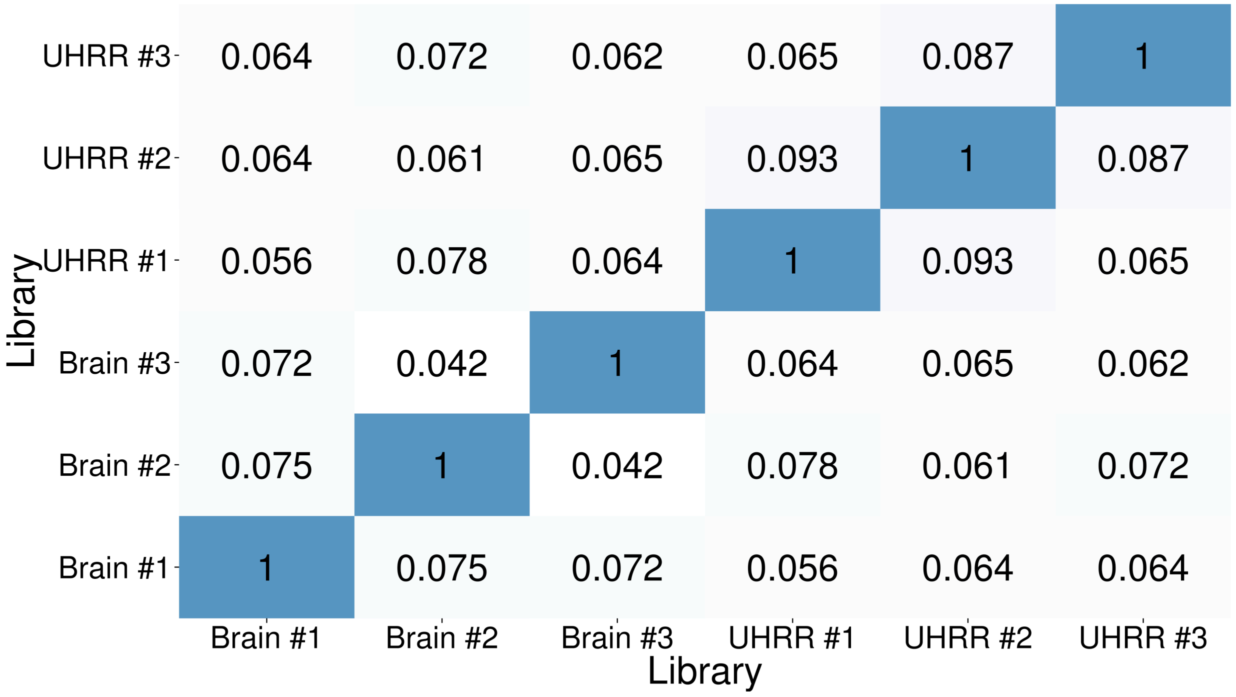

Supplementary Figure S4. Barcode correlation between paired-end EXB groups. A Pearson correlation coefficient between replicates of the number of reads found in each paired-end EXB barcode group is performed for 10ng of input cDNA. A low correlation coefficient indicates that specific EXB sequences are not preferentially amplified.

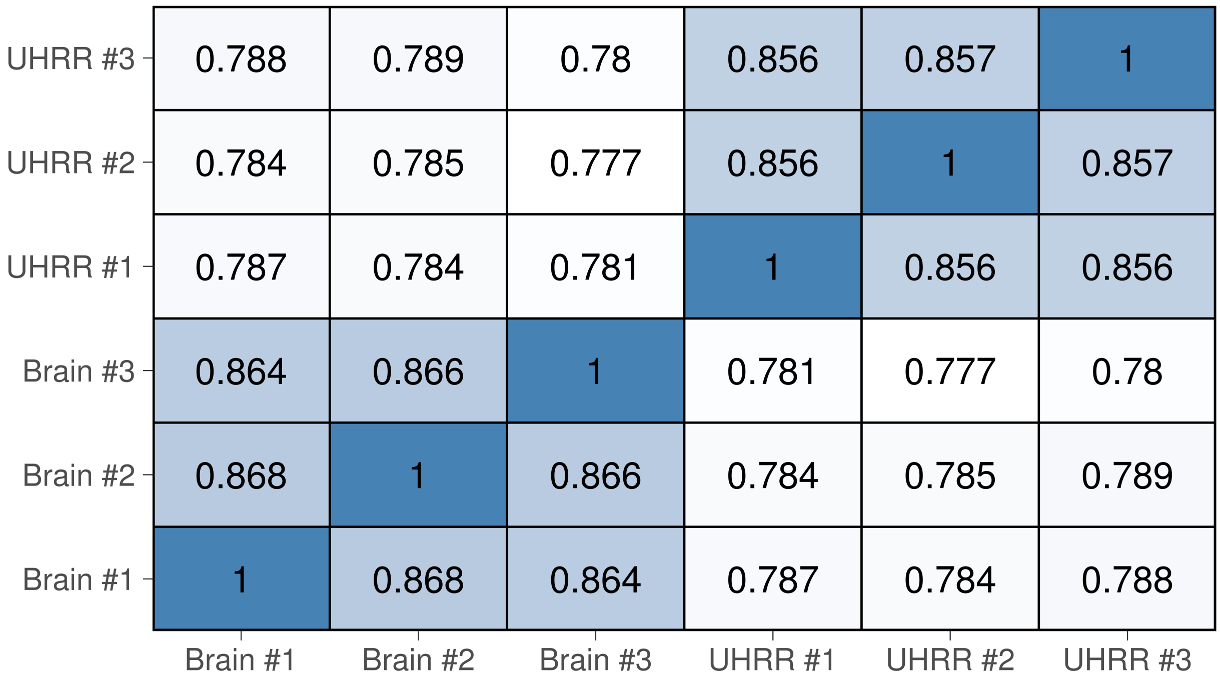

Supplementary Figure S5. Gene expression correlation between EXB-labelled transcriptome data at 10ng input. The Spearman correlation coefficients of EXB-based expression counts between different MAQC samples at 10ng input cDNA is shown.

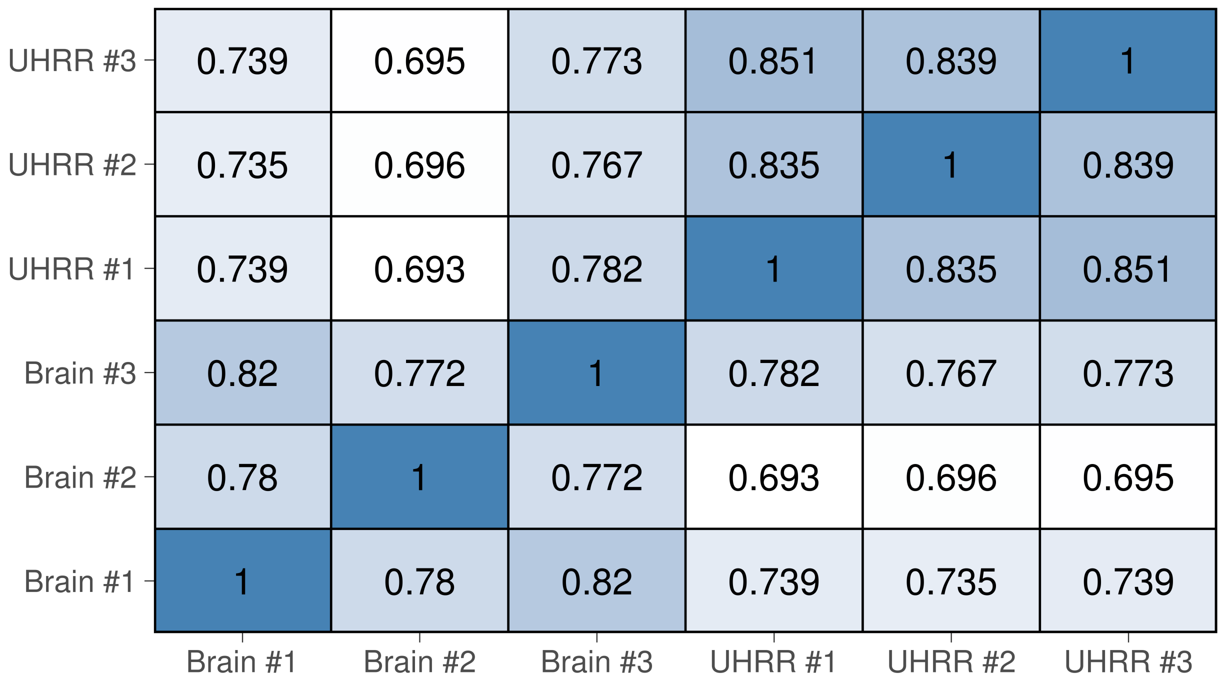

Supplementary Figure S6. Gene expression correlation between EXB-labelled transcriptome data at 1ng input. The Spearman correlation coefficients of EXB-based expression counts between different MAQC samples at 1ng input cDNA is shown.

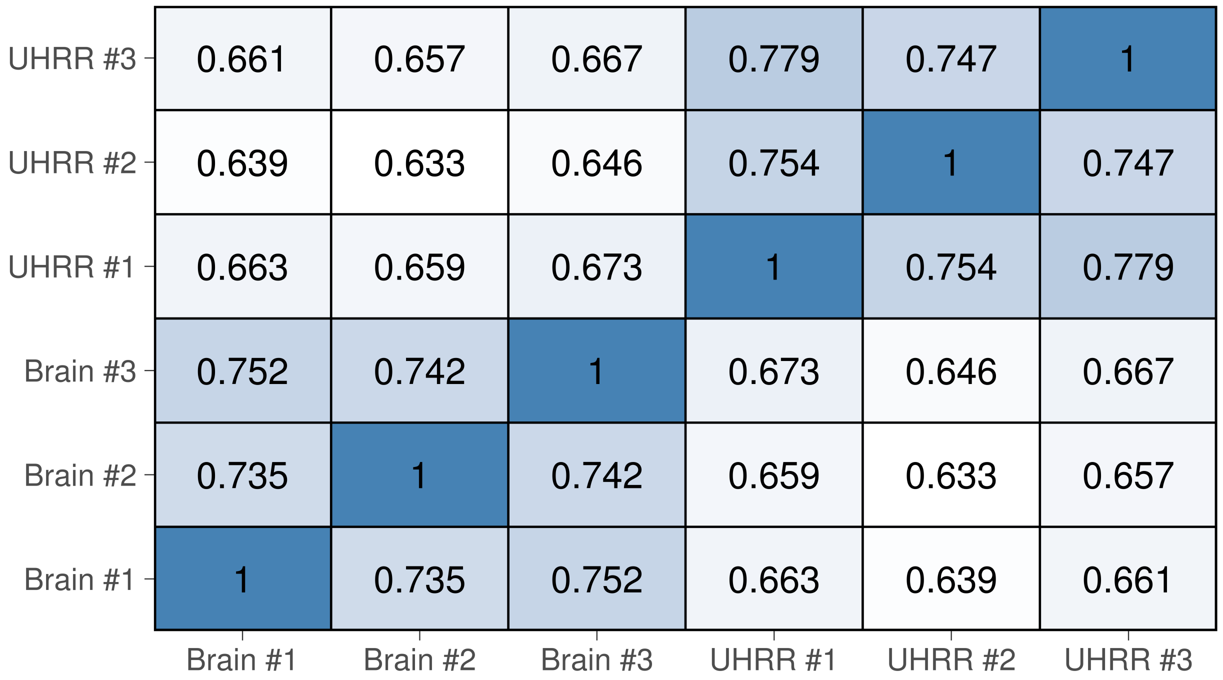

Supplementary Figure S7. Gene expression correlation between EXB-labelled transcriptome data at 100pg input. The Spearman correlation coefficients of EXB-based expression counts between different MAQC samples at 100pg input cDNA is shown.

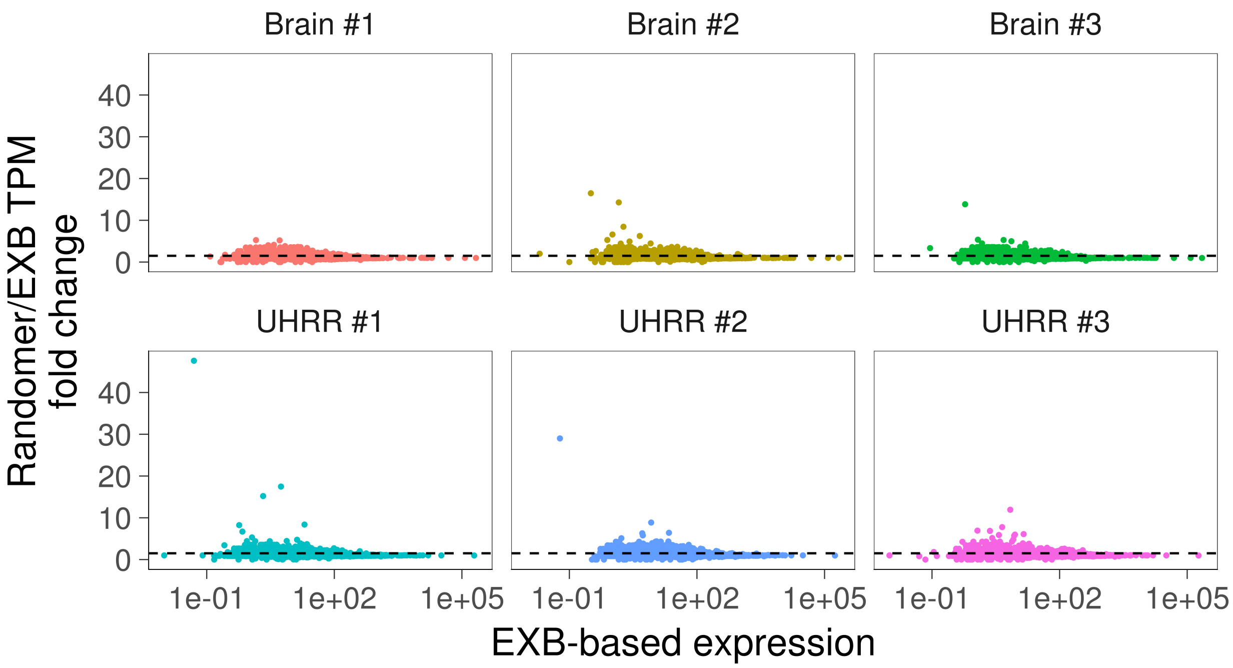

Supplementary Figure S8. Artifactual effects of random-mer-based expression analysis as a function of EXB expression level. The fold-change between random-mer and EXB-based transcriptome quantification is shown for all experiments with 100pg input cDNA. A higher fold-change indicates a larger discrepancy between the quantification methods. A dashed line showing a threshold fold-change of 1.5-fold between methods is shown.

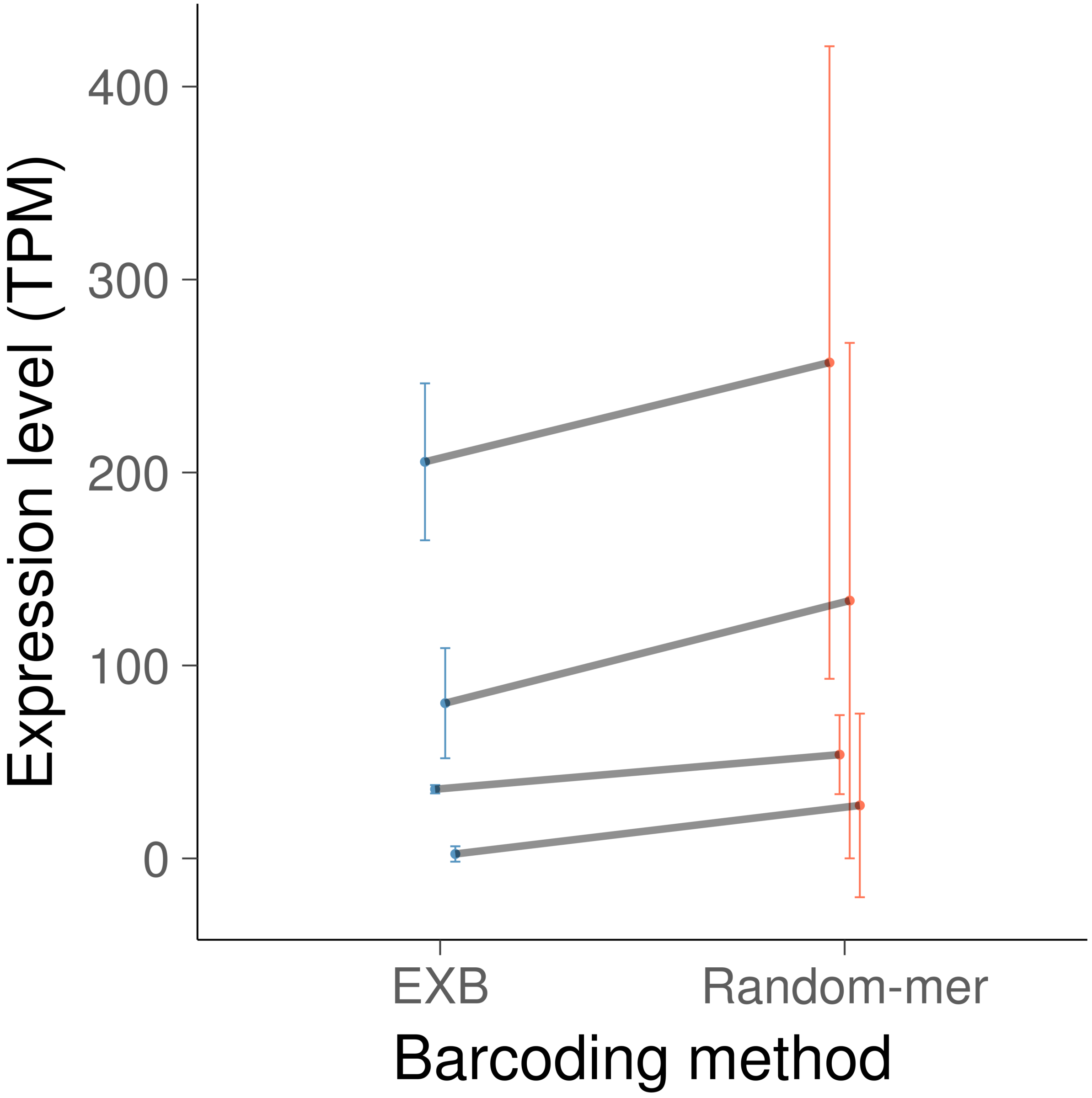

Supplementary Figure S9. Technical variation between counting methods. Several genes were chosen (ENSG00000257342, ENSG00000163171, ENSG00000166037, ENSG00000044115) in the 100pg brain cDNA dataset to demonstrate the change in technical variability when counting with random-mer molecular barcodes.

Supplementary Table S1. List of barcodes generated by linear coding matrix.

AAAAAA	CAACCC	GAAGGG	TAATTT
AACCTG	CACGAT	GACTCA	TACAGC
AAGGGA	CAGTTC	GAGAAG	TAGCCT
AATTCG	CATAGT	GATCTA	TATGAC
ACACGT	CCAGTA	GCATAC	TCAACG
ACCGCC	CCCTGG	GCCATT	TCCCAA
ACGTAT	CCGACA	GCGCGC	TCGGTG
ACTATC	CCTCAG	GCTGCT	TCTTGA
AGAGAG	CGATCT	GGAAGA	TGACTC
AGCTTA	CGCAAC	GGCCCG	TGCGGT
AGGAGG	CGGCTT	GGGGAA	TGGTCC
AGTCCA	CGTGGC	GGTTTG	TGTAAT
ATATGC	CTAATG	GTACAT	TTAGCA
ATCACT	CTCCGA	GTCGTC	TTCTAG
ATGCAC	CTGGCG	GTGTGT	TTGATA
ATTGTT	CTTTAA	GTTACC	TTTCGG

Supplementary Table S2. List of oligonucleotides. As separate file.

Supplementary Table S3. Sequencing statistics for 10ng input cDNA assays.

	MAQC 10ng					
**Sample info**	10ng Brain #1	10ng Brain #2	10ng Brain #3	10ng UHRR #1	10ng UHRR #2	10ng UHRR #3
**Raw reads**	29.38M	33.52M	29.81M	30.64M	31.42M	32.99M
**Barcode grouping:**						
**Reads with EXB passing filter**	21.25M	24.86M	23.24M	23.33M	23.39M	24.67M
**# unique EXB groups passing filter**	16.32M	18.22M	16.47M	17.27M	18.25M	18.66M
**# EXB groups with non-unique randomer**	0.07M	0.10M	0.09M	0.08M	0.08M	0.09M
**RNA-Seq alignment of barcode groups:**						
**# EXB groups mapped by STAR**	15.82M	17.70M	16.06M	16.82M	17.72M	18.14M
**# EXB groups uniquely mapped**	11.54M	12.88M	11.57M	12.26M	12.87M	13.24M
**% EXBs with duplicate genome alignment**	23.6%	25.2%	26.6%	23.3%	21.8%	22.5%
**# EXB groups multi-mapped**	4.28M	4.82M	4.50M	4.56M	4.85M	4.90M

Supplementary Table S4. Sequencing statistics for 1ng input cDNA assays.

	MAQC 1ng					
**Sample info**	1ng Brain #1	1ng Brain #2	1ng Brain #3	1ng UHRR #1	1ng UHRR #2	1ng UHRR #3
**Raw reads**	17.06M	12.49M	31.23M	27.46M	17.53M	21.28M
**Barcode grouping:**						
**Reads with EXB passing filter**	11.04M	8.04M	13.65M	15.60M	11.74M	13.94M
**# unique EXB groups passing filter**	2.14M	1.23M	5.82M	4.85M	2.20M	2.94M
**# EXB groups with non-unique randomer**	0.13M	0.09M	0.18M	0.19M	0.14M	0.18M
**RNA-Seq alignment of barcode groups:**						
**# EXB groups mapped by STAR**	1.51M	0.87M	3.87M	3.46M	1.66M	2.18M
**# EXB groups uniquely mapped**	0.99M	0.56M	2.31M	2.24M	1.12M	1.45M
**% EXB groups with duplicate genome alignment**	50.4%	53.4%	44.8%	45.9%	50.0%	49.8%
**# EXB groups multi-mapped**	0.51M	0.31M	1.56M	1.22M	0.53M	0.73M

Supplementary Table S5. Sequencing statistics for 100pg input cDNA assays.

	MAQC 100pg					
**Sample info**	100pg Brain #1	100pg Brain #2	100pg Brain #3	100pg UHRR #1	100pg UHRR #2	100pg UHRR #3
**Raw reads**	25.99M	23.41M	24.63M	24.07M	21.09M	24.14M
**Barcode grouping:**						
**Reads with EXB passing filter**	8.45M	7.90M	8.10M	8.48M	7.08M	8.51M
**# unique EXB groups passing filter**	2.48M	2.24M	2.55M	2.42M	1.69M	2.13M
**# EXB groups with non-unique randomer**	0.10M	0.09M	0.09M	0.10M	0.08M	0.10M
**RNA-Seq alignment of barcode groups:**						
**# EXB groups mapped by STAR**	1.16M	1.03M	1.16M	1.16M	0.75M	1.04M
**# EXB groups uniquely mapped**	0.62M	0.55M	0.61M	0.65M	0.44M	0.60M
**% EXB groups with duplicate genome alignment**	46.7%	49.2%	47.3%	48.4%	49.6%	49.4%
**# EXB groups multi-mapped**	0.55M	0.48M	0.55M	0.51M	0.32M	0.43M

Supplementary Table S6. Comparison of MAQC qPCR and EXB-based quantification methods. As separate file.

Supplementary Table S7. Comparison of MAQC qPCR fold-change and EXB-based fold-change quantification methods. As separate file.

Supplementary Table S9. Comparison to other molecular barcoding sequencing methods.

**Study**	**This study**	**Shiroguchi et al.**	**Fu et al.**	**Kivoja et al., Islam et al., Brennecke et al., Macosko et al., Klein et al.**	**Kinde et al.**	**Schmitt et al. x2**
**Sample type**	RNA<br>Full length	RNA<br>Full length (*E. coli*)	RNA<br>Full length	RNA<br>5' or 3' ends	DNA	DNA
**Number of barcodes**	69 billion	21k	960	1024-65,536	16-268 million	2.8 trillion
**Barcode design**	Pre-designed; forward engineered	Randomly designed then pairwise comparison	Pre-designed	Random	Random	Random
**Incorporation method**	Reverse transcriptase; transposase	Ligation	Ligation	Reverse transcriptase	DNA polymerase	Ligation
